# The advancement of a gender ethics protocol to uncover gender ethical dilemmas in midwifery: a preliminary theory model

**DOI:** 10.1186/s12978-022-01515-6

**Published:** 2022-11-19

**Authors:** Monica Christianson, Sine Lehn, Marianne Velandia

**Affiliations:** 1 Department of Nursing, Umeå University, 901 85 Umeå, Sweden; 2 Institut for Mennesker Og Teknologi (Department of People and Technology), Roskilde Universitet, Universitetsvej 1, Bygn. 44.1, 4000 Roskilde, Denmark; 3 School of Health, Care and Social Welfare, Division of Caring Science, Midwifery Program, Mälardalen University, Högskoleplan 1, Box 883, 721 23 Västerås, Sweden

**Keywords:** Ethics, Feminist approach, Gender, Midwifery, Method development, Theory model

## Abstract

**Objectives:**

Ethical dilemmas at both the individual and structural level are part of the daily work of midwives and gender inequality and injustice can affect women’s sexual and reproductive health. Mainstream bioethical theory has been criticized for neglecting women’s issues. To ensure women’s experiences are addressed, a gender lens on ethics is crucial.

**Aim:**

This study develops a theory model by exploring ethical dilemmas related to gender in the context of maternity care from the perspective of midwifery science and feminist ethics.

**Methods:**

The research strategy followed a coherent stepwise approach: literature search, thematic analysis, elaboration of a gender ethics protocol, and the integration of various components into a preliminary gender ethics model for midwifery.

**Findings:**

A literature search was performed using Scopus and Web of Science to identify ethical dilemmas in maternity care linked to gender and power. The search of articles published between 1996 and 2019 returned 61 abstracts. These abstracts were screened and assigned one of the following themes: The Midwifery Profession, The Rights of the Woman, Fetal Rights Dominate, and Medicalization of Pregnancy and Childbirth. A tentative gender ethics frame was developed and tested on two articles on abortion, one from Denmark and one from Japan. The protocol facilitated the gender analysis of ethical dilemmas related to abortion, which were related to the imbalance of power relations in health care. In the final step, we synthesized the dimensions of gender and power in a gender ethics model for midwifery.

**Discussion:**

The gender ethics protocol developed revealed gendered dimensions of ethical dilemmas in midwifery. This gender analysis adds to the understanding of the “do no harm” principle by revealing assumptions and stereotypes that promote unequal power relations. The gender ethics model is an innovative approach that envisions and exposes power imbalance at the micro, meso, and macro levels.

**Conclusions:**

The protocol could improve gender competence among researchers, midwives/professionals, and midwifery students throughout the world.

## Plain Language summary

As gender inequity, gender inequality, and oppression infuse dimensions in all human cultures and societies, not the least in midwifery practice where the layers of injustice affect women during pregnancy and birth in high-, middle-, and low-income countries, the time has come to renew the perspectives of normative ethics. In this study, we explore gender ethical dilemmas unique to maternity care from the perspective of midwifery science and feminist ethics. A literature search uncovered ethical dilemmas in midwifery, and four broad themes were identified: Midwifery Profession, Rights of the Woman, Fetal Rights Dominate, and Medicalization of Pregnancy and Childbirth. Next, we developed a gender ethics protocol suitable for providing gender ethical interpretations of results. The protocol was tested and refined using two articles (one from the Denmark and one from Japan) that address ethical dilemmas of abortion care. The pilot analysis indicates that the autonomy of midwives and their scope of practice might be constrained and that the obstetric medicalized/authoritative knowledge still plays a dominate role in maternity practice. Here, we present an elaborate model, gender ethics model for midwifery (GEMM), developed for midwifery science that can be further refined. The model challenges the views of maternity care and contributes to a deeper understanding of how fluid concepts such as gender and power circulate and influence women’s and birthing person’s sexual and reproductive health.

## Statement of significance

- Problem or issue
  Layers of injustice affect women during pregnancy and birth in both high-, middle-, and low-income countries. The time has come to renew the perspectives of normative ethics.
- What is already known?
  Globally, ethical dilemmas at both the individual and structural levels pervade the daily work of midwives, but ethical research that includes gender theory in midwifery science is limited.
- What this paper adds
  Here, we present a gender ethics protocol and develop an innovative gender theory ethics model for midwifery to improve gender competence among researchers, midwives/professionals, and midwifery students.

## Introduction

Globally, ethical dilemmas at both the structural and individual level pervade the daily work of midwives [1]. Structural dilemmas include lack of access to sexual and reproductive health, unfair distribution of health care resources, and discrimination of girls and women. Every day, midwives face several individual dilemmas when caring for women with drug problems or seeking an abortion, including the expectation that they will be whistleblowers when health care is harmful [1]. Oppression and gender inequity and inequality infuse all human cultures and societies, not the least in midwifery practice, where the layers of injustice affect women during pregnancy and birth in both high-, middle-, and low-income countries [2]. In low-income countries, both midwives and women are affected by high maternal mortality rates; in middle- and high-income countries, both midwives and women are affected by unnecessary surgery and technical and pharmacological interventions in childbirth. The time has come to renew the perspectives of normative ethics. To the best of our knowledge, ethical research that includes gender theory in midwifery science has been limited. This lack of focus forms the starting point for our method. As we could not find a sustainable model for our purpose, we describe the steps used to develop a theoretical gender ethics model (GEMM) in midwifery science.

### Codes of ethics and the lack of gender ethics

After World War II, renewed research ethics codes were developed for various research areas [3]. In 1947, the Nuremberg Code, the first modern ethics code, was established to prevent human experimentation abuses. This code stated what researchers should do during the research process, from informed consent to publication to archiving material. The code contributed to creating praxis protocols and increased awareness of potential ethical problems in research. Rules and concepts from the Declaration of Helsinki, adopted by the World Medical Association in 1964 (revised in 2013), are central to research in medicine and health. In 1990, the World Health Organization (WHO) established guidelines for epidemiological research in low- and middle-income countries (i.e., research in populations and communities with limited resources): WHO International Guidelines on Ethics and Epidemiology (followed by WHO International Guidelines for Biomedical Research Involving Human Subjects, 1993). These guidelines emphasize local solutions rather than paternalism.

Theories of ethics are important in many sectors of society, including midwifery science. Ethical perspectives pervade all parts of professional midwifery and research. The International Confederation of Midwives (ICM), American College of Nurse-Midwives (ACNM), Midwives Alliance of North America (MANA), and National Association of Certified Professional Midwives (NACPM) provide the basic ethical codes for professional midwives globally [4]. These codes of ethics address broad ethical principles in medicine, such as respect for autonomy, non-maleficence, beneficence, and rights [3].

As the current foundation of ethics focuses on ensuring human rights, it aligns with gender ethics. However, there is a gap in knowledge about gender equality and embedded power dimensions.

### Feminist ethics and midwifery

The ethical codes of conduct for midwives are an integral part of the midwifery profession, where the major principles—professional competence, informed consent, respect for privacy, respect for diversity, respect for women’s values, and respect for women’s self-esteem—can be synthesized and framed as pro-women [4]. From a feminist point of view, these traditional westernized ethical principles have been criticized for being male-centric as they rely on individualism and agency, rationality, and independence [5], preferences that could mask power relations, dominance, and structural inequalities. From an ethicist’s perspective, these preferences disregard women’s experiences and interests [6] and disrespect women’s capabilities for moral reflection [7].

Midwifery is a gendered profession: most midwives identify as women and care for “other” women’s sexual and reproductive health during the whole life course. However, most midwives still have less power compared to male doctors across cultures as an androcentric view has dominated childbirth [2]. Globally, when midwifery moved from the private domain of homebirths to the public sphere of institutional births (i.e., obstetric care at hospitals), obstetric risk discourse emerged [5], which endangers the normality of pregnancy and birth by pathologizing pregnancy and birth. Consequently, when the practice moved into institutional childbirth, the midwives’ cultural identity became restricted by “technocratic” procedures, where many interventions comprise and engrain the (non)experience of normal birth, rather than supporting “natural” births [8, 9]. The midwifery social model of care, informed choice, and the relational approach to autonomy are often in stark contrast to a technocratic and medicalized model of birth, where the bioethics of informed consent and its idea of autonomy dominate [10]. However, the medical model of informed consent only partly maintains and respects the autonomy of the birth-giving woman, as it may also preserve “paternalistic predecessors” (e.g., women/patients who do not “obey” medical advice are perceived as noncompliant) [10].

Jaggar [11], criticizing mainstream bioethical theory, proposed moral guidelines that address neglecting “women’s issues” and devaluating women’s moral experiences. From her perspective, the traditional western ethics devalue women’s interests and rights, suggesting that women are less morally developed than men. Culturally, masculine traits such as independence, autonomy, and rationality are overvalued, whereas feminine traits such as interdependence, childbearing, emotion, are undervalued. Finally, mainstream bioethical theory and proposed moral guidelines favor culturally masculine ways of moral reasoning, which emphasize rules, universality, and impartiality, at the expense of culturally feminine ways of moral reasoning, which emphasize relationships, particularity, and partiality [11].

For Jaggar [11], a feminist approach to ethics seeks to do the following:

- articulate moral critiques of actions and practices that perpetuate women’s subordination;
- prescribe morally justifiable ways of resisting such actions and practices;
- envision morally desirable alternatives for such actions and practices; and
- consider women’s moral experiences seriously although not uncritically.

Feminist ethics have informed our development of a gender ethics model for midwifery (GEMM), and the theoretical framework reflects our preunderstandings. Acknowledging that this framework takes a European or Anglo-American understanding of gender norms as a base does not mean that we are reifying the West. Discrimination and misogyny are universal issues. Our point of departure is that ethical research has not included gender theory in midwifery science. In this article, we focus on ethical dilemmas in sexual and reproductive health that affect midwives and the women they treat.

## Objective

Our overall aim was to develop a theory model to explore the gender ethical dilemmas unique to maternity care from the perspective of midwifery science and framed by feminist ethics. Specifically, we wanted to develop a gender ethics protocol suitable for analyzing ethical dilemmas and test and refine the gender ethics protocol using two articles about abortion.

## Methods

A literature review was used to identify the available evidence in the field and to point out the contradictions and gaps in the existing knowledge [12]. The first and the last author performed a comprehensive search on the topic and developed the steps for the search [13]. Several approaches have been used to conduct systematic reviews in various areas of health where general topics and concepts such as health care interventions, genetic associations, and policy making are perceived to be relevant to any systematic review [13]. Hence, modifications are sometimes required depending on the research. Here, we modified the Prisma flow chart to fit our purpose and topic.

### Literature search

Our search strategy focused on two multidisciplinary databases—Scopus and Web of Science. The Scopus database covers more than 20,000 journals, most from Medline and PubMed, that include caring and social science perspectives. Scopus is the largest abstract and citation database of peer-reviewed literature, which includes scientific journals, books, and conference proceedings. Web of Science includes approximately 12,000 journals, partly unique and partly overlapping journals at Scopus. A librarian at Umeå University assisted in the search. First, we made a broad search in Scopus using the search term Midwife*AND Ethics* for all years up to 2016. This search returned 809 articles. Most of the articles were not specifically about midwives and ethics. Therefore, a search was performed using the search terms Midwife*AND Ethical Dilemmas* for all years. This search returned 58 articles. A second search was performed in Scopus for the years 2017 and 2018, which returned eight articles. To improve the consistency across the report of the review, we refined the search at Web of Science (2019) and found 23 articles.

### Inclusion and exclusion criteria

**Fig. 1:**
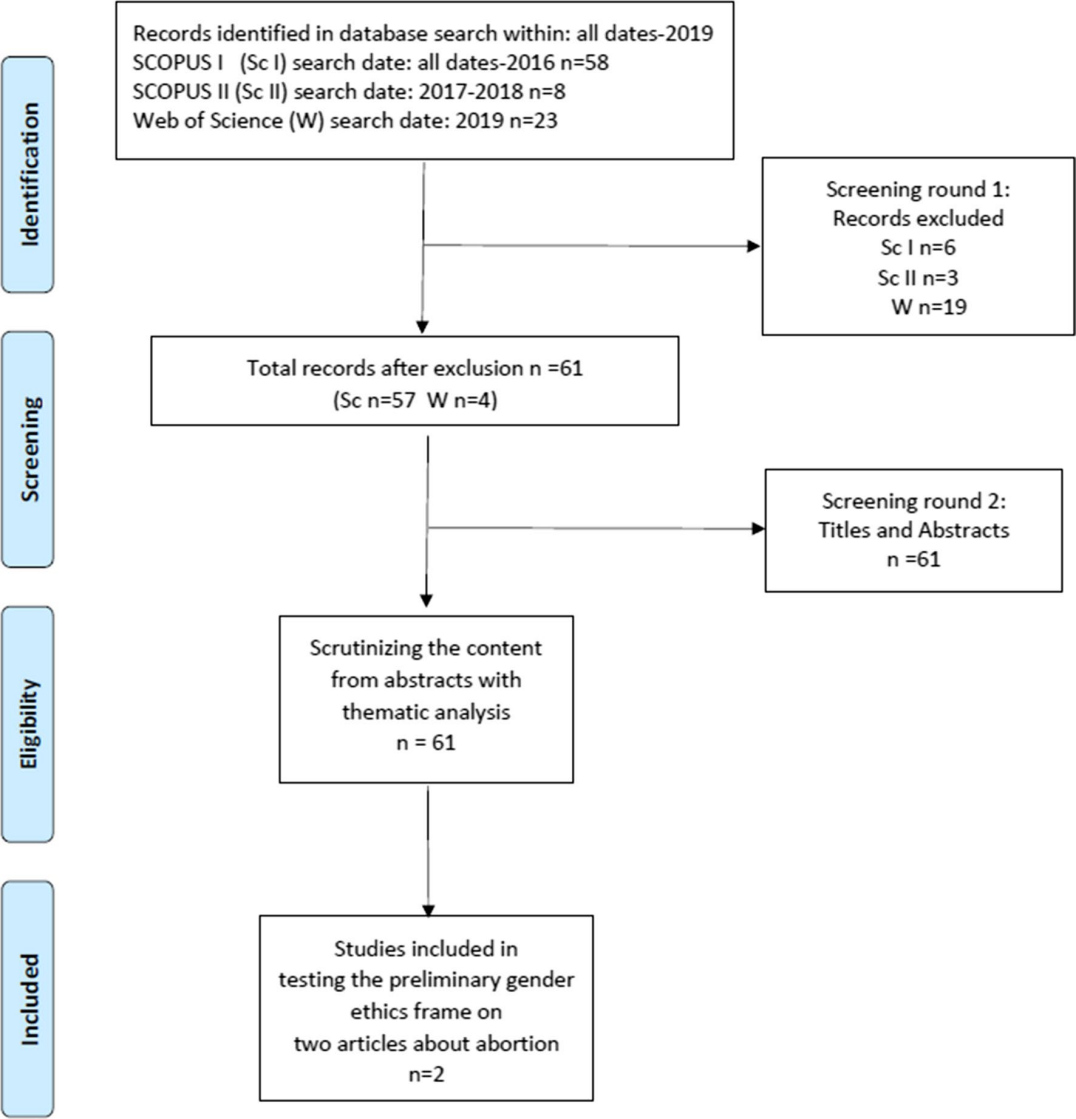
PRISMA flow diagram of the literature search and development of gender ethics frame

Peer-reviewed quantitative and qualitative articles from the perspectives of midwifery not before 1995 were included. Nine articles from the Scopus searches were excluded: two articles about nursing perspectives, one article with a missing abstract, three articles published before 1995, two articles not in English, and one not relevant for midwifery. When we compared the articles from the Web of Science search with the articles from the Scopus search for duplicates, we found four abstracts in Web of Science that were not published in Scopus. The search also identified 23 articles from the Web of Science of which 17 were duplicates. In addition, one article was excluded because it was in German and one was excluded because it was a review. Therefore, 19 articles in total were excluded. The four abstracts were read and the articles were included. We included the suggested steps in accordance with the different phases identified by Moher et al. [13], except for the two last steps—i.e., qualitative synthesis and meta-analysis of quantitative studies. Therefore, 61 abstracts composed the basis for a qualitative analysis inspired by thematic analysis according to Braun and Clarke [14]. Figure 1 shows a flow chart that overviews the literature search.

## Results

**Fig. 2:**
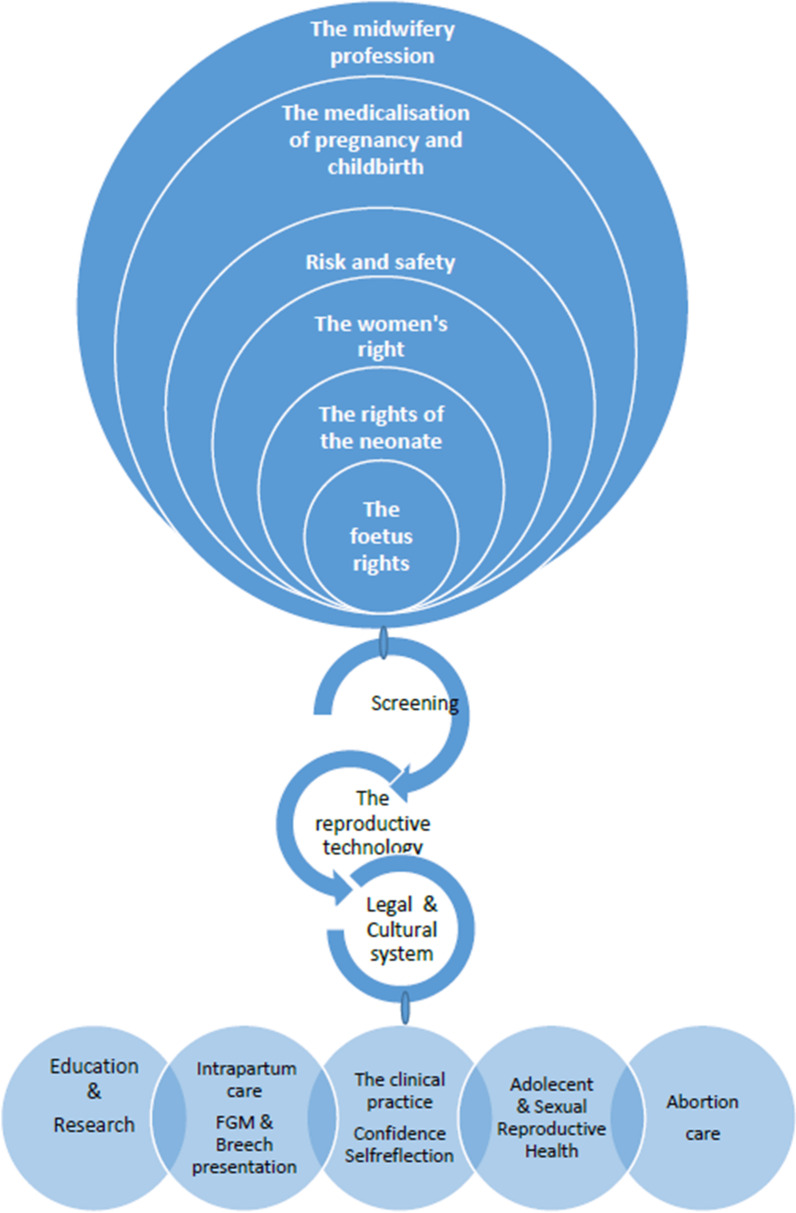
Mind map with examples of preliminary themes and subthemes evolved from scrutinizing the abstract content

The literature search, which included articles published between 1996 and 2019, returned 61 abstracts. We performed a thematic analysis of the abstracts using Braun and Clarke’s six phases: reading and coding the abstracts, developing preliminary themes, discussing the preliminary themes, finding consensus about themes, and naming the final themes [14]. We created a mind map inspired by Braun and Clarke [14] and the following preliminary themes emerged: Midwifery Profession; Medicalization of Pregnancy and Childbirth; Risk and Safety; Women’s Rights; Rights of the Neonate; and Fetus Rights. There were eight preliminary subthemes (Fig. 2). We further refined the analysis and re-sorted the preliminary themes in the final analysis. The names of final themes and subthemes are presented in Table 1. In total, 26 articles concerned the midwifery profession. We identified 14 articles as Women’s Rights, eight articles as Fetus Rights, and 13 articles as Medicalization of Childbirth, which includes articles on reproductive technologies, screening, and testing.

**Table 1:** Themes and subthemes and included articles from the search

Themes	Subthemes	Number of articles in each theme In total n = 61**
Midwifery Profession	Education for students Education for professionals (midwives/physicians) Midwifery science research FGM Abortion care	1, 4, 6, a7, a8, 10, 11, 13, 15, 18, 19, 24, 26, 27, 31, 32, 38, 39, 47, 49, 51,54, Sc a1, a5, W4, W5 **n = 26**
Rights of the woman	Conflicts between maternal and fetal rights	2, 8, 16, 9, 20, 21, 23, 28, 29, 40, 42, 46, 48, W20 **n = 14**
Fetal rights dominate	The rights of the fetus/neonate	3, 5, 7, 22, 34,35,37, 45 **n = 8**
Medicalization of pregnancy and childbirth	Breech Risk and safety Reproductive technology Prenatal screening Genetic testing	12, 14, 17, 25, 30, 33, 36, 41, 43, 44, 50 Sca6, W12 **n = 13**

*Sc* first Scopus search, *Sca* second Scopus search, *W* Web of Science

*Each included article received a number

**Appendix of the 61 articles for abstract analysis

### To develop a gender ethics protocol—a suitable tool for analyzing ethical dilemmas

The gender perspective in ethics research is limited. In the recommendations from The 2030 Agenda for Sustainable Development [15], a gender analysis could, for example, focus on fairness and needs, distribution and access to resources, decision-making, or power relations between women and men. Another merit of a gender analysis is the possibility to detect if there is a need for specific targeted actions for women and girls (in line with our project). Moreover, a gender analysis adds to the understanding of the ‘do no harm’ principle by challenging assumptions and stereotypes that reproduce unequal power relations.

### Research triangulation during the development of the gender ethics protocol

During a 2-day workshop in Denmark, the research group performed and developed all analytical steps together for credibility and validity reasons in line with investigator triangulation [16]. Two of the researchers are midwives, one with experience working at maternity care and one with experience working at youth clinic and the third researcher has a background in sociology and her research field covers professionalism, learning and practice in healthcare. These opposing perspectives—i.e., midwifery science, gender, and social sciences—revealed how the various aspects about gender (sometimes different/sometimes overlapped) were open for discussion, which strengthened our standpoints and increased our knowledge. In concrete terms, we discussed various gender concepts and reached consensus about which items to include in the preliminary gender ethics frame. In this elaboration, we created a draft frame with perceived important items to visualize multiple gender and power dimensions. The following power dimensions were discussed: East versus West perspectives, local versus world perspectives, and the inclusion of a multitude of voices [17]. Moreover, we elaborated the context of the study and the critical reflection about who is doing the research, who is being researched, what is being researched, how is research being done, where is research being done, and why research is being done [18–20]. We identified the consequences of the complex combination of how various power dimensions and oppressions are captured such as how privileged versus unprivileged is in line with an intersectional approach [21]. Furthermore, we identified research in professional power dynamics and the gendered and power-permeated nature of professionalized maternity praxis [22, 23]. As a final analytical move, we focused on silenced content in the articles (i.e., what is not there/not talked about with relation to gender and power) [24]. In the next step, we applied the tentative gender ethics protocol in a pilot study (Table 2).

**Table 2:** The gender ethics protocol

Article 1	Article 2
1. Ethical dilemmas presented in the article Overarching ethical dilemma: Whether the fetus shows sign of life after termination of pregnancy Underlying ethical dilemma: The increasing influence of other professions, especially physicians, becomes apparent in the counselling of pregnant women/couples after diagnosis of fetal abnormalities	1. Ethical dilemmas presented in the article Overarching ethical dilemma: That Japanese midwives are obliged to assist women with abortion and/or that they are poorly educated to take on this task Underlying ethical dilemma: Midwives cannot decline abortion care Abortion and deliveries are carried out in the same institutional framework
2. Context of the study Midwives in Denmark working with late termination of pregnancy (TOP) Danish TOP practices	2. Context of the study Abortion care education programs in nurse and midwifery schools in Japan. Abortion is legal up to week 21 for reasons such as rape, the physical health of the mother, or socioeconomic hardship Most of the abortions are performed before gestational week 12 Medical abortions using drugs are rare in Japan. Surgical methods such as dilatation and curettage are used
3. Method (e.g., qualitative/quantitative/observation/document analyses) Qualitative interviews with ten midwives	3. Method (e.g., qualitative/quantitative/observation/document analyses) A descriptive study to determine the extent of abortion care education programs and the respondents’ perceptions of abortion care education
** (**a) Who is researching what The researchers from the unit of women and gender research in medicine, Department of Public Health	(a) Who is researching what A researcher from the Division of Health Care Science, Kanazawa University, Japan
(b) Empirical material (data) Interview transcripts with midwives	(b) Empirical material (data) A questionnaire was developed based on ten topics identified form an analysis of 220 textbooks about reproductive health
** (**c) Preunderstanding of the researchers No preunderstanding is stated	** (**c) Preunderstanding of the researchers No preunderstanding is stated
4. Theory? (e.g., feminist analysis, content analysis, grounded theory) Grounded theory (Corbin and Strauss), Theories of professions (Brante and Eriksen)	4. Theory? (e.g., feminist analysis, content analysis, grounded theory) None
5. The article’s conclusion about the ethical dilemmas The personification of the fetus is driven by the changing guidelines at hospitals The views of the professionals, the wishes of the women/couples, and the influence of other professionals. (gynecologists/obstetrics) in “nagging” the freedom for midwives in their work with women/couples	5. The article’s conclusion about the ethical dilemmas Education and practice guidelines should be developed Abortion care education and training for nurses and midwives in Japan have deficits. Abortion care must be incorporated Abortion care education and training are lacking in education and training programs The skills in reproductive health such as family planning and abortion care should be in the curricula for midwives as the midwives are required to treat women in a professional manner (the code of conduct)
6. Gender Women/couples. Midwives = predominantly women How is gender conceptualized? Gender = “sex” (men and women) Gender as a social category (how they act and react) Gender as a power relationship (e.g., disagreement between pregnant women, physicians, or midwives/obstetricians) Different positions in the hierarchy where physicians influence midwives Gender and power: If physicians do not order enough medication during the abortion, midwives will be alone and responsible for taking care of the dying fetus when parents refrain refuse Midwives disagreed with how physicians counselled the women/couples after prenatal “diagnosis.” Further research is needed to secure the best possible working conditions for midwives and how to optimize the care for women/couples The institutional setting is highly influenced by other professionals (physicians)	6. Gender 93% of responders were women How is gender conceptualized? Gender = “sex” (men and women) Gender as a social category (how they act and react) Gender as a power relationship (e.g., disagreement between pregnant women, physicians, or midwives/obstetricians) Disagreement concerning abortion care. The author indicates that in the US and in many EU countries health care providers can decide if they want to take part in the abortion Male pressure (disagreement?) on a woman to undergo an abortion has a negative impact on women’s mental health The obstetrician/gynecologist’s power to decide the abortion method and choose a more harmful method instead of a modern method for abortion (mifepristone) Imbalanced power in decision making between obstetricians and midwives The lack of mandate for midwives in relation to abortion care is a serious shortcoming
** (**a) Gender as invisible Gender/power is visible in the article. The structure of the organization indicates that doctors are making decisions rather than midwives	(a) Gender as invisible Gender is invisible. Although the WHO recommends medical abortion, most women in Japan must have a surgical abortion with curette dilatation
7. Power dynamics in the article (e.g., East/West, privileged/unprivileged,) Midwives are a female profession that differentiates from “the medical focus that is otherwise present at the hospital and among other professions.” Power dynamics—YES! Midwives are less privileged compared to doctors = hierarchy, a female job	7. Power dynamics in the article (e.g., East/West, privileged/unprivileged) As midwives are supervised and employed by obstetricians, midwives cannot decline the routines (the sharp curette method)
(a) Acceptable, unacceptable practices? (e.g., not acceptable to leave midwives with a fetus that is still alive) “Midwives experience losing the ability to set the conditions for their own work [...]. Instead they choose to focus on their own professional identity and hold on to their ideals, even though it is difficult at the hospital” (918) Not acceptable that midwives should be responsible for handling a dying fetus “The increasing influence of physicians becomes apparent in the counselling of pregnant women/couples after diagnosis of fetal abnormalities. [...] Several midwifes are critical of the counselling sessions. They believed that physicians had already made a decision as to whether or not to perform late TOP before speaking to the woman/couple” (917–18)	** (**a) Acceptable, unacceptable practices? (e.g., misogyny or pro-women centered) Not acceptable that health care providers can decline abortion care
** (**b) Who does the article talk about as the “good” professional and who is the “other” = othering, e.g., fat women as deviating from the norm? The midwives are perhaps the “good” professionals	** (**b) Who does the article talk about as the “good” professional and who is the “other” = othering, e.g., fat women as deviating from the norm? Difficult to say: nurses? midwives? East/West (midwives in US and EU are allowed to refuse to take part in an abortion)
8. What is not talked about in the article What other professions or the women think What is not talked about: The reason why midwives must handle the dying fetus: Is it their responsibility? Men/fathers or partners are not discussed in the article	8. What is not talked about in the article Authors relationship to the field of research How do women experience abortion services? Is abortion care contradictory to midwifery? Women’s situations in abortion care are not talked about The costs (financial, psychological, and physical) Ethical issues are there but rarely discussed Anti-feminist approaches

Focus of data extracts and analysis from two articles on ethical dilemmas in abortion care

### Test and refine the gender ethics protocol using two articles from the literature search

To test the effectiveness of the gender ethics analysis, we used abortion as a subtheme in two articles, one from Denmark (Article 1) and one from Japan (Article 2), as abortion is an important ethical topic for both women and midwives. WHO and ICM recommends that midwives (and sometimes nurses) be the key providers of abortion services [25, 26]. From our viewpoint, the chosen topic has relevance for the midwifery profession. Each of us separately performed an analysis of the whole content of the two test articles. The tentative gender frame was applied to each study. During a research meeting in Sweden, we compared our individual analysis and discussed and re-sorted the analysis until consensus was achieved. We performed the analysis, first individually and then in a group session, to avoid biases (e.g., single observer bias) [16]. See Table 2 for a data extraction of the analysis.

## Result

The application of the gender ethics protocol added value by improving our analysis of gendered dimensions, confirming that this approach could be very useful for researchers in midwifery science (Table 2).

In both articles, we found an overarching ethical dilemma along with underlying dilemmas. When researchers are exploring ethical dilemmas, the presentation of the ethical dilemmas must be clearly stated. Both articles blurred these topics although they were less visible in article 2. The context was not well described, which is a pitfall of much of the research in medicine and health [27]. We could easily see what methods the researchers had used. Article 1 also referred to theories, whereas article 2 did not. The item “who is researching what” informs whether researchers are part of the context they are investigating. We believe that it is important that empirical research address whether researchers from the West are exploiting or marginalizing the East [17] as this poses the risk for paternalism. This was not seen in the articles. Moreover, neither article identified the researchers’ preunderstandings, which is a drawback as this would have ensured quality and broadened the view of research [28]. Identifying preunderstandings is an important aspect as the motive for investigating ethical issues may bias the analysis and interpretation of results. Neither article used gender or feminist theory. From our point of view, using gender or feminist theory could have improved the quality of the content from an epistemological and ontological perspective, which might have made the articles’ conclusions even stronger. Gender and power relations were overt in both articles. According to our analysis, this points towards the finding that the biomedical framing of abortion silences its gendered nature. Both articles pointed to the professional hierarchies within the health care system and the tensions between midwives and physicians. For example, in article 1, the author stated that “midwives have unique knowledge of the feelings of a woman/couple going through late TOP and can contribute valuable insight into the field. However, midwives were critical of how physicians counsel women/couples after prenatal diagnosis.” In addition, the midwives requested more freedom to organize the care as midwives, compared to physicians, have a more holistic view of the women’s/couples’ situation when it comes to deciding about TOP. Other professions as well as structural factors at the hospital influenced the midwives’ ability to organize their work with late terminations. In Japan, the midwives must deal with opposing care—assisting the delivery or assisting the abortion. This conflict between personal convictions and professional duties can make it difficult to maintain emotional control. The change of role from midwife to abortion care provider causes a dilemma or crisis of professional identity, especially for new midwives. Furthermore, it is ethically questionable whether a woman seeking an abortion should be placed in the same setting as post-partum women and newborn babies [27].

As gender dynamics and power relations seem to be interwoven and should not be seen as separable, we suggest that those dimensions be analyzed together. The issue of privileged versus unprivileged is part of an intersectional analysis and is well suited for this method [18]. Research about both gender and professionalism needs to define acceptable or unacceptable practices to ensure respectful and ethical practices are maintained (e.g., a code of ethics) as well as to discuss who is considered a “good” professional and who is not [29]. In Japan, midwives work under the direction of an obstetrician or gynecologist, cannot prescribe contraceptive drugs, and cannot refuse abortion care service. Because of the lack of abortion care in Japanese curricula, modern methods of abortion care are not used. As 97% of the early abortions in Japan are surgical, women are exposed to undesirable practices, leading to non-evidence-based methods and a form of obstetric violence. Midwifery education should ensure that future midwives receive sufficient skills in abortion care. In Japan, midwifery education undermines the midwife’s position and role in abortion care, so it contributes to weakening or suppressing an abortion-seeking woman’s position in her own health care.

This is an important aspect as the risk for othering and ethnocentric vision at times are gender-blind issues. Finally, investigating what was not talked about proved interesting. From a discursive perspective, these issues are highly relevant [30], and we identified several dimensions that we thought would have been important to elicit especially in article 2 (Table 3).

**Table 3:** Overview of the suggested dimensions in the tentative gender ethic protocol

x) Author, Title of the article, Year of publication, Journal, Title of the authors (e.g., midwives, physicians, sociologists, and psychologists) [20]	
1. Ethical dilemmas presented in the article (e.g., fairness, needs, distribution, and access to resources) [15, 21]	
2. Context of the study (e.g., hospital in DK) (example micro, meso, macro level [17, 27]	
3. Methodology (e.g., qualitative/quantitative/observation/document analysis) (a) Who is researching what? [18, 19] (b) Empirical material (data) (c) Preunderstanding of the researchers [28]	
4. Theory (e.g., feminist analysis, content analysis, and grounded theory) [6–8, 10, 11]	
5. The article’s conclusion about the ethical dilemmas [7, 11, 19]	
6. Gender (a) How is gender conceptualized? [17, 18, 21] (b) Gender = “sex” (men, women, or not defined as either/or) (c) Gender as a social category (how human beings act and react), “race,” binary gender (d) Gender as power relation (Can we find disagreement between, e.g., pregnant women, physicians, or midwives/obstetricians, decision-making and power relations between women and men?) (e) Gender as invisible	
7. Power dynamics in the article (a) ( e.g., East/West, privileged/unprivileged, discrimination [19] (b) Acceptable/unacceptable practices? (e.g., not acceptable to leave midwives with a fetus that is still alive) [27] (c) Who does the article talk about as the “good” professional, who is the “other” = othering, e.g., fat women for deviating from the norm [29]	
8. What is not talked about in the article [24, 30]	

^*^Examples of useful references

### Presentation of gender ethics model for midwifery, GEMM

**Fig. 3:**
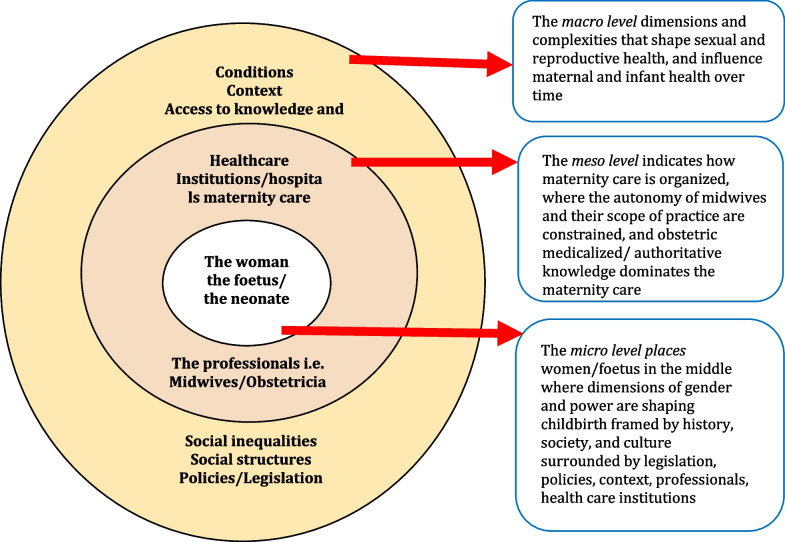
The gender ethics model for midwifery, GEMM

In Fig. 3, we present an elaborate model developed for midwifery science, GEMM. The identified themes presented in Table 1 and criteria for the gender ethics protocol are integrated in the preliminary model. The model challenges the traditionally assumed views of maternity care, which focuses on obstetric risk discourses [5]. That is, the model contributes to a deeper understanding of how fluid concepts such as gender and power circulate and influence women’s and birthing person’s sexual and reproductive health concretely. In each circle of the model, we provide examples of individual organisational and structural dimensions that affect sexual and reproductive health and childbirth. These dimensions are condensed and included in the GEMM in line with social theory [31].With inclusion of feminist ethics [7, 10, 11], we can easily imagine how the micro, meso, and macro levels perpetuate women’s subordination historically, culturally, and contemporaneously in patriarchal societies. From our point of view, the gender ethics protocol adds to the understanding of gender theory and improves the epistemological and ontological dimensions in midwifery science by eliciting novel questions, debating moral critiques of practices that perpetuate women’s/birthing person’s subordination, resisting and describing justifiable or alternative views in line with feminist ethics, addressing informed choice in full, and respecting both pregnant women and birthing women/person’s autonomy. This approach values women’s/birthing person’s moral experiences as interdependence, body, emotion, and nature, emphasizing relationships, particularity, and partiality. However, the inclusion of gender theories not only uncovers what we interpret as obstacles in maternity care but also adds value, as awareness and deeper knowledge among researchers in midwifery science may lead to a renewed focus on equity and equality changes in sexual and reproductive health.

## Results and discussion

We discovered a gap in knowledge concerning the ability to understand, uncover, and synthesize ethical dilemmas in midwifery from a gender perspective in published research articles. As we could not find a sustainable model suitable for our purpose, we developed a gender ethics model for midwifery to understand the dynamic relations of gender and power and how these are produced and reproduced and to develop alternative courses of action in line with ethics of care [7, 10, 11].

From the literature search, inspired by thematic analysis, four broad themes were identified and named: Midwifery Profession, Rights of the Woman, Fetal Rights Dominate, and Medicalization of Pregnancy and Childbirth. We were identified, selected, and critically appraised the research that we found relevant for the overall aim: to explore the gender ethical dilemmas unique to maternity care and unpack these as a starting point for developing a gender ethics protocol suitable for providing gender ethical interpretations of results. This protocol was further refined and used in our pilot study about abortion. The protocol enabled a gender analysis of the two pilot articles. The actual outcome of the pilot analysis indicates that the autonomy of midwives and their scope of practice might be constrained and that obstetric medicalized/authoritative knowledge still plays a dominate role in maternity practice. For example, in Japan, the old curette dilation method is still used during abortions although medical abortion is proven to be a safer, cheaper, and ethically desirable alternative. A retrospective study from Japan published in 2015 [32] showed that only 0.3% of the abortions were medical, and abortion with curette and *sharp* curette dominated. According to the WHO [25], medical abortions should be easily accessible, safe, effective, and acceptable in both high- and low-resource settings and resources should be used efficiently. Many interventions in medical abortion care, particularly those in early pregnancy, can now be provided at the primary care level and on an outpatient basis, which further increases access to care. Medical abortion care reduces the need for skilled surgical abortion providers and offers a non-invasive and highly acceptable option for pregnant individuals [25]. In Denmark, as midwives are exposed to viable fetuses after abortions and supervised by obstetricians, they can experience conflicts between their personal and professional values. Therefore, it is important that midwives in Denmark be exposed to morally justifiable practices in line with feminist ethics [11]. The subordination of women by men and the subordination of midwives by the medical profession are maintained despite decades of political efforts to support equity and gender equality. Our analysis suggests that the imbalance in power relations between obstetricians and midwives contributes to the emergence of ethical dilemmas. Interestingly, in both articles, we see significant misogyny and discrimination, pointing to the universality when it comes to women, equity, and equality. Above, we have articulated actions and practices that preserve women’s subordination. A recent literature review by Fleming et al. [33] identifies the many reasons why midwives and nurses should not participate in abortion care and abortion remains a morally contentious issue with some midwives and nurses [33]. Fleming et al. state that the International Confederation of Midwives’ Code of Ethics supports this stance as “midwives may decide not to participate in activities for which they hold deep moral opposition” [33], although this does not address how the necessary care should be provided. Whereas the WHO’s recent guidelines on abortion [25] do not address the issue of conscientious objection, the ICM affirms that “a woman who seeks or requires abortion-related services is entitled to be provided with such services by midwives” [26]. The gender ethics protocol was tested and refined during the analysis of one qualitative and one quantitative article of the subtheme abortion. The analysis revealed interesting tensions between midwives and obstetricians. In addition, the analysis suggests that the refusal of midwives to take part in abortions may be framed as a gender ethical problem in the West as refusal by a midwife could restrict women’s access to abortion.

Finally, GEMM can be used to synthesize dimensions of gender and power with theoretical underpinnings and ultimately be used as to elaborate these dimensions. GEMM can also be used to visualize how gender and power are produced and reproduced and help GEMM reveal gender ethics issues. Gender analysis can add to the understanding of how gender inequities/inequalities are produced and reproduced in a given situation or praxis such as maternity care and how inequalities can be changed [21].

In this article, we systematically presented the various steps taken to develop a gender ethics model for midwifery, GEMM. To address our research question, we combined a traditional and “a modified” literature review in line with Moher et al.’s recommendations [13] with a gender analysis inspired by feminist ethics of care [7, 8, 10, 11]. Although a systematic review usually requires including many articles, this study focused on a gender ethics model so the risk for excessive reviews addressing the same question is probably very close to zero. In the literature search, we identified, selected, and critically appraised the research that we found relevant for the study aim. This was a starting point for developing a gender ethics protocol suitable for providing gender ethical interpretations of results from articles. Here, we have delineated a stepwise process of method development around ethics in midwifery science using a gender lens. To address our research questions and develop the gender ethic model for midwifery, we took inspiration from prestigious gender researchers in the field [17–19] and ethics of care [7, 10, 11]. By combining a modified systematic literature search and Moher et al.’s recommendations [13], we retrieved abstracts mentioning ethical dilemmas in maternity care and scrutinized these using a thematic analysis approach developed by Braun and Clarke [14].

## Strengths and limitations

Scopus and Web of Science were searched during the data collection, and these large databases cover more than 20,000 and 12,000 journals, respectively, which is a strength. The search in selected journals in the field of midwifery science was performed to ensure that specific articles about ethical dilemmas in maternity care were covered. Despite this effort, we might have missed relevant articles. We could have performed a new search of articles published since 2019 as new publications will always turn up; however, we judged that limiting the search to 23 years was sufficient. Transparency guided the research process and the various methodological steps were described in the development of the gender ethics model, an audit trail that the reader can follow [34]. To increase the validity and credibility of the findings, we combined a modified systematic literature search, a qualitative thematic analysis, and gender theory as using a variety of methods, according to Noble and Heale [35], provides a more balanced explanation for readers. In addition, investigator triangulation can increase validity [16]. To perform a comprehensive understanding of elaborating a model for ethics and gender in midwifery, we used data collection methods that best fit our research questions. Being a group of three researchers representing different backgrounds strengthened the gender analysis. However, a researcher’s background and perspective can influence how texts are interpreted. Researchers with other perspectives will probably have another interpretation. Research triangulation helped achieve trustworthiness and transference, and the research team were all immersed in the data [14]. The development of the GEMM benefited from our broad interdisciplinary experiences from health care science, midwifery science, and social science, our specific gender competence, our experiences with methods, our experiences with midwifery (two of the authors), and our professional experience outside midwifery (one author). In a future study, we will further develop and use the gender ethics protocol to explore gender and ethics in midwifery science.

## Conclusion

There is limited ethical research that uses gender theory to explore midwifery science. This model is a starting point for advancing this area of research. To our knowledge, this is the first protocol in gender ethics to explore ethical dilemmas in midwifery science. In this paper, we look at midwifery science literature from a perspective of feminist ethics to develop a theory model that describes gender ethical dilemmas. To do this, we employed a modified systematic literature review and a thematic analysis to elaborate a gender ethics frame and integrate the individual to structural components in the gender ethics model for midwifery (GEMM). From the broad literature search, inspired by thematic analysis, four broad themes were identified. The gender ethics protocol was tested and refined during the analysis of one qualitative and one quantitative article from the subtheme abortion. Two articles about abortion/TOP in midwifery are not enough to arrive at a universally applicable theory, but it is a solid beginning. In low- and middle-income countries, access to abortion care is sometimes restricted or hidden and information lacking [36]. Often, illegal drug sellers, people working with illegal providers, sex workers, taxi drivers, or feminist groups are key informants in the search for abortion providers. Globally, women are dying because of unsafe abortions; however, midwives can provide respectful evidenced-based abortion care [37]. In the final stage of developing GEMM, we visualized how gender and power are produced and reproduced. Consensus was reached that the GEMM helped uncover ethical issues related to gender.

As gender researchers, we know that there is an overt risk that gender dimensions are ignored if researchers are unwilling to admit the existence of power imbalance in the lives of humans, if researchers lack the specific gender competence, or if researchers lack an adequate methodological tool. The protocol and the GEMM represents a strategy to develop a method for gender analysis that can be used in research and education. When using the gender ethics protocol, we recommend including researchers with gender competence as this will advance the gender analysis.

## Further research

In a next article, we plan to apply the gender ethics protocol to a major set of articles from a 5-year exploration of ethical dilemmas and gender issues in midwifery practice and research to use and further validate the strengths and eventual limitations of the gender ethics model.

## Data Availability

We have included an Appendix Matrix to provide an overview of the articles included.

## Appendix: Overview of the articles included

**Table:**

Total (n = 61)	Theme	Author, year, title, journal, article number
(n = 26)	*Midwifery profession*	Bedwell C, et al. 2012, Using diaries to explore midwives’ experiences in intrapartum care: an evaluation of the method in a phenomenological study. *Midwifery*. (15) Hays KE, et al. 2015, The Professionalization of International Disaster Response: it is time for midwives to get ready. *Journal of Midwifery and Women’s Health*. (4) King T, 1997, Epidural anesthesia in labor: benefits versus risks, *Journal of Nurse-Midwifery*. (51) Narchi NZ, de Castro et al. 2017, Report on the midwives’ experiences in the Brazilian National Health System: a qualitative research. *Midwifery*. (a5) Narrigan D, 2004, Examining an ethical dilemma: a case study in clinical practice, *Journal of Midwifery and Women's Health. (38)* Pezaro S, 2018, Confidentiality, anonymity and amnesty for midwives in distress seeking online support—ethical?, *Nursing Ethics*. (Sca1) Polona Mivšek et al. 2015, How do midwives in Slovenia view their professional status? *Midwifery* (1) Vinggaard Christensen A, et al. 2013, Faced with a dilemma: Danish midwives’ experiences with and attitudes towards late termination of pregnancy, *Scandinavian Journal of Caring Sciences*. (11) Wilson-Mitchell K, et al. 2016, Infusing diversity and equity into clinical teaching: training the trainers, *Journal of Midwifery and Women’s Health*. (a7) Woodward VM, 1998, Caring, patient autonomy and the stigma of paternalism, *Journal of Advanced Nursing*. (49)
	Education for students	Buxton M, et al. 2015, Simulation: a new approach to teaching ethics, *Journal of Midwifery and Women’s Health* (6) Church S, et al. 2013, Student midwives’ responses to reproductive ethics: a qualitative focus group approach using case scenarios, *Midwifery* (13) Geraghty SM, et al. 2009, An Australian perspective: the art of being with students, *Journal of Continuing Education in Nursing.* (26) Karline Wilson-Mitchell et al. 2016, Infusing diversity and equity into clinical teaching: training the trainers*, **J Midwifery Womens Health*. (W4) Klingberg-Allvin M, et al. 2007, Ethics of justice and ethics of care. Values and attitudes among midwifery students on adolescent sexuality and abortion in Vietnam and their implications for midwifery education: a survey by questionnaire and interview, *International Journal of Nursing Studies.* (32) Megregian, Michele, 2016, Ethics education in midwifery education programs in the United States, *J Midwifery Womens Health*. (W5) Mizuno M, 2014, Abortion-care education in Japanese nurse practitioner and midwifery programs: a national survey, *Nurse Education Today* (10) Scheilling SM, et al. 1994, Everyday ethics and nurse/midwifery education, *Nurse Education Today*. (54)
	Education for professionals	Anderson BA, et al. 2007, Simply not there: The impact of international migration of nurses and midwives-perspectives from Guyana, *Journal of Midwifery and Women's Health.* (31) Comerford Freda M, 2004, Issues in patient education, *Journal of Midwifery and Women's Health*. (39) Houwink EJ, et al. 2011, Genetic educational needs and the role of genetics in primary care: a focus group study with multiple perspectives, *BMC Family Practice* (18) Megregian M, 2016, Ethics education in midwifery education programs in the United States, *Journal of Midwifery and Women's Health*. (a8) Mngadi PT, et al. 2008, Health providers’ perceptions of adolescent sexual and reproductive health care in Swaziland, *International Nursing Review.* (27) Webb J, et al. 1999, Getting it right: the teaching of philosophical health care ethics, Nursing Ethics. (47)
	Midwifery science research	Ryan K, et al. 2011, Which hat am I wearing today? Practising midwives doing research, *Evidence Based Midwifery* (19)
	FGM	Hess RF, et al. 2010, Knowledge of female genital cutting and experience with women who are circumcised: a survey of nurse-midwives in the United States, *Journal of Midwifery and Women’s Health*. (24)
(n = 14)	*The rights of the woman*	Brodrick A, 2014, Too afraid to push: dealing with fear of childbirth. *The Practising Midwife* (8) Dotters-Katz S, et al. 2014, Cancer and pregnancy: the clinician’s perspective, *Obstetrical and Gynecological Survey*. (9) Jankowski J, et al. 2015, Home birth of infants with congenital anomalies: a case study and ethical analysis of careproviders’ obligations. *The Journal of Clinical Ethics* (2) Lowe NK, 2004, Context and process of informed consent for pharmacologic strategies in labor pain care, *Journal of Midwifery and Women’s Health*. (40) Pickering, D. 2011, A fight for the right to life, *Practising Midwife*. (16) Rees J, 2011, A consideration of ethical issues fundamental to researching sensitive topics: substance use during pregnancy, *Evidence Based Midwifery.* (20) Sharp ES, 1998, Ethics in reproductive health care: a midwifery perspective, *Journal of Nurse-Midwifery*. (48) Thompson, Faye E, 2003, The practice setting: site of the ethical conflict for some mothers and midwives, *Nursing Ethics*. (W20)
	Conflicts between maternal and fetal rights	Burrows J, 2001, The parturient woman: can there be room for more than ‘one person with full and equal rights inside a single human skin’? *Journal of Advanced Nursing*. (46) Cignacco E, 2002, Between professional duty and ethical confusion: midwives and selective termination of pregnancy, *Nursing Ethics.* (42) Cunningham FG, et al. 2010, NIH consensus development conference draft statement on vaginal birth after cesarean: new insights. *NIH consensus and state-of-the-science statements*. (23) Dann L, 2007, Grace’s story: an analysis of ethical issues in a case of informed consent, *British Journal of Midwifery*. (28) Garel M, et al. 2007, Ethical problems of extreme prematurity: results of a qualitative study with obstetricians and midwives | [Problèmes éthiques posés par l'extrême prématurité: résultats d'une étude qualitative auprès des obstétriciens et des sages-femmes], *Gynecologie Obstetrique Fertilite.* (29) Ledward A, 2011, Informed consent: ethical issues for midwife research, *Evidence Based Midwifery*. (21)
(n = 8)	*The rights of the fetus/neonate*	Avci E, 2015, Caregivers’ role in maternal–fetal conflict. *Narrative Inquiry in Bioethics* (3) Bream KDW, et al. 2005, Barriers to and facilitators for newborn resuscitation in Malawi, Africa, *Journal of Midwifery and Women's Health*. (34) Crawford D, et al. 2010. The care of the pre-viable and questionably viable infant—a midwife and a neonatal nurse dilemma*, Journal of Neonatal Nursing*. (22) Eyre TA, et al. 2015, Management and controversies of classical Hodgkin lymphoma in pregnancy, *British Journal of Haematology.* (5) Garel M, et al. 2004, Ethical decision-making for extremely preterm deliveries: results of a qualitative survey among obstetricians and midwives, *Journal of Maternal–Fetal and Neonatal Medicine*. (37) Kenner C, et al. 2005, Newborn screening and genetic testing, *Journal of Midwifery and Women's Health*. (35) Pritchard I, 2015, Using Principlism to resolve the ethical dilemma of withdrawing treatment from a neonate diagnosed with Spinal Muscular Atrophy, *Journal of Neonatal Nursing*. (7)
	Children’s rights	Charles-Edwards I, et al. 2002, Ethics and children’s rights: learning from past mistakes. *British Journal of Nursing* (Mark Allen Publishing). (45)
(n = 13)	*The medicalization of pregnancy and childbirth*	
	Breech	Walker S, 2013, Undiagnosed breech: towards a woman-centred approach, *British Journal of Midwifery*. (14)
	Risk and safety	Minkoff H, et al. 2013, A reconsideration of home birth in the United States, *Journal of Clinical Ethics* (12)
	The reproductive technology	Burrage J, 1998, Infertility treatment in women aged over 40 years. Nursing standard (Royal College of Nursing (Great Britain): 1987). (50) Papaharitou S, et al. 2007, Reproductive health and midwives: does occupational status differentiate their attitudes on assisted reproduction technologies from those of the general population? *Human Reproduction.* (30)
	Prenatal screening	Edvardsson K, et al. 2016, Increasing possibilities—increasing dilemmas: a qualitative study of Swedish midwives’ experiences of ultrasound use in pregnancy, *Midwifery*. (Sca6) Garel M, et al. 2002, Ethical decision-making in prenatal diagnosis and termination of pregnancy: a qualitative survey among physicians and midwives, *Prenatal Diagnosis*. (43) Gottfredsdóttir H, 2010, ‘Have You Had the Test?’ A discourse analysis of media presentation of prenatal screening in Iceland, *Scand J Caring Scie*. (W12) Hundt GL, et al. 2011, Inside 'Inside View': Reflections on stimulating debate and engagement through a multimedia live theatre production on the dilemmas and issues of pre-natal screening policy and practice, *Health Expectations* (17) Pinard JA et al. 2003, Maternal serologic screening for toxoplasmosis, *Journal of Midwifery and Women's Health*. (41) Reid B, et al. 2009, A meta-synthesis of pregnant women's decision-making processes with regard to antenatal screening for Down syndrome, *Social Science and Medicine*. (25) Williams C, 2006, Dilemmas in fetal medicine: premature application of technology or responding to women’s choice? *Sociology of Health and Illness*. (33)
	Genetic testing	Lea DH, et al. 2005, Ethical issues in genetic testing, *Journal of Midwifery and Women’s Health*. (36) Williams C, et al. 2002, Is nondirectiveness possible within the context of antenatal screening and testing? *Social Science and Medicine*. (44)

## Abbreviations

GEMM: Gender ethic model for midwifery
ICM: The International Confederation of Midwives
WHO: World Health Organization
